# Hyperinvasive approach to out-of hospital cardiac arrest using mechanical chest compression device, prehospital intraarrest cooling, extracorporeal life support and early invasive assessment compared to standard of care. A randomized parallel groups comparative study proposal. “Prague OHCA study”

**DOI:** 10.1186/1479-5876-10-163

**Published:** 2012-08-10

**Authors:** Jan Belohlavek, Karel Kucera, Jiri Jarkovsky, Ondrej Franek, Milana Pokorna, Jiri Danda, Roman Skripsky, Vit Kandrnal, Martin Balik, Jan Kunstyr, Jan Horak, Ondrej Smid, Jaroslav Valasek, Vratislav Mrazek, Zdenek Schwarz, Ales Linhart

**Affiliations:** 1 2nd Department of Medicine - Department of Cardiovascular Medicine, 1st Faculty of Medicine, Charles University in Prague and General University Hospital in Prague, U Nemocnice 2, Prague 2, 128 00, Czech Republic; 2 Emergency Medical Service Prague, Korunni 98, 101 00, Prague 10, Czech Republic; 3 Institute for biostatistics and analysis, Masaryk University Brno, Kotlářská 2, Brno, Czech Republic; 4 Department of anesthesiology, resuscitation and intensive medicine, 1st Faculty of Medicine, Charles University in Prague and General University Hospital in Prague, U Nemocnice 2, Prague 2, 128 00, Czech Republic

**Keywords:** Cardiac arrest, Hypothermia, Extracorporeal life support, Mechanical compression device, Invasive assessment

## Abstract

**Background:**

Out of hospital cardiac arrest (OHCA) has a poor outcome. Recent non-randomized studies of ECLS (extracorporeal life support) in OHCA suggested further prospective multicenter studies to define population that would benefit from ECLS. We aim to perform a prospective randomized study comparing prehospital intraarrest hypothermia combined with mechanical chest compression device, intrahospital ECLS and early invasive investigation and treatment in all patients with OHCA of presumed cardiac origin compared to a standard of care.

**Methods:**

This paper describes methodology and design of the proposed trial. Patients with witnessed OHCA without ROSC (return of spontaneous circulation) after a minimum of 5 minutes of ACLS (advanced cardiac life support) by emergency medical service (EMS) team and after performance of all initial procedures (defibrillation, airway management, intravenous access establishment) will be randomized to standard vs. hyperinvasive arm. In hyperinvasive arm, mechanical compression device together with intranasal evaporative cooling will be instituted and patients will be transferred directly to cardiac center under ongoing CPR (cardiopulmonary resuscitation). After admission, ECLS inclusion/exclusion criteria will be evaluated and if achieved, veno-arterial ECLS will be started. Invasive investigation and standard post resuscitation care will follow. Patients in standard arm will be managed on scene. When ROSC achieved, they will be transferred to cardiac center and further treated as per recent guidelines.

**Primary outcome:**

6 months survival with good neurological outcome (Cerebral Performance Category 1–2). Secondary outcomes will include 30 day neurological and cardiac recovery.

**Discussion:**

Authors introduce and offer a protocol of a proposed randomized study comparing a combined “hyperinvasive approach” to a standard of care in refractory OHCA. The protocol is opened for sharing by other cardiac centers with available ECLS and cathlab teams trained to admit patients with refractory cardiac arrest under ongoing CPR. A prove of concept study will be started soon. The aim of the authors is to establish a net of centers for a multicenter trial initiation in future.

**Ethics and registration:**

The protocol has been approved by an Institutional Review Board, will be supported by a research grant from Internal Grant Agency of the Ministry of Health, Czech Republic NT 13225-4/2012 and has been registered under ClinicalTrials.gov identifier: NCT01511666.

## Introduction

Cardiac arrest (CA) is a significant socio-economic burden [1,2]. The aim of the care for patients suffering from cardiac arrest is a neurologically intact survival, ie, avoidance of irreversible organ damage, mainly the brain hypoxic-reperfusion injury. However, neurologically favourable survival in patients resuscitated worldwide by emergency services is only 5–15%, eventually 8–40% in patients with initially shockable rhythms [3]. In Prague, in 2008, 493 patients were resuscitated by Prague Emergency Medical Service (EMS) for OHCA (out of hospital cardiac arrest). ROSC (return of spontaneous circulation) was reached in 56% of cases, 43% survived the episode, 15% were discharged home with favourable neurological outcome, however, back to the fully active life including job attendance returned only 7% of the original cohort [4]. A key prerequisite for a successful outcome is minimalization of time delays, resuscitation quality, complex intensive care and treatment of cardiac arrest cause [5-7]. So far, the only proven method for increased survival with good neurological outcome is early initiation of mild hypothermia and probably also the rapidly reached target temperature [3,8]. However, the use of hypothermia affects individual estimation of prognosis [9,10] and the whole topic of hypothermia needs further evaluations and studies including potentially beneficial intraarrest cooling [11-15]. Recent systematic review on intraarrest hypothermia confirmed its beneficial effect in terms of survival and neurological outcome in an experimental setting, however, clinical data on the efficacy of intraarrest cooling are still limited [16-20].

Similarly, chest compression devices are being increasingly used in OHCA, despite the fact that their role is still controversial [21-25]. They provide uninterrupted continuous compressions even during the transport, decrease the demands on EMS crew and provide a bridge to other methods like PCI (percutaneous coronary intervention) or ECLS (extracorporeal life support)/ECMO (extracorporeal membrane oxygenation) initiation [26,27]. Current European Resuscitation Council (ERC) guidelines [26] consider mechanical compression devices, ie, LUCAS (Lund University Cardiac Arrest System; Physio-Control Inc./Jolife AB, Lund, Sweden) and Autopulse (LDB - load distributing band; ZOLL, Chelmsford, MA, U.S.A.) to be potentially beneficial, however, with not yet evidently proven beneficial impact on patients survival and recommend further randomized studies.

Accordingly, the indication of extracorporeal life support devices during cardiopulmonary resuscitation (CPR) is controversial in CA patients and no definitive role has been determined. Encouraging results of ECLS for CA of cardiac origin in adults were shown recently both for IHCA (inhospital cardiac arrest) and OHCA [28-33], in inhospital pediatric CA [34-36] and recently has been even proposed for out of hospital “on scene” refractory CA [37-39]. However, the results are still not satisfactory yielding wide survival rate range from 4% [32] to 48% [33]. This may be related to different definitions of refractory CA, ie, from 10 [31] to 30 minutes [33] before ECLS initiation is considered. For in hospital CA the survival with good neurological outcome has been observed in up to 20 to 42% of cases [26,28,30,40,41]. Therefore, ECLS has been assigned a low-grade recommendation in recent guidelines for inhospital cardiac arrest [42]. However, the good results obtained in IHCA cardiac arrests can not be automatically extrapolated to OHCA patients because of longer transport times and possible delay in ECLS initiation [43].

Therefore, we designed a randomized trial of “hyperinvasive approach” encompassing all above mentioned sophisticated methods and hypothesized, that changed logistics of prehospital refractory OHCA management and immediate on-admission ECLS institution might bring beneficial impact on patient survival [44].

Assuming, that refractory cardiac arrest may be caused by a treatable condition, all mentioned interventions are approached as only temporizing techniques to allow for further diagnostics and therapy, mainly the coronary angiography ± PCI, eventually other investigations and treatments (i.e. pulmonary angiography and catheter embolectomy, aortography or brain computed tomography, CT).

The aim of this comparative study is to collect prospective, randomized data on prehospital use of a chest compression device combined with intraarrest evaporative cooling as a bridge to in hospital emergency ECLS implantation followed by immediate invasive diagnostics and treatment in cases of witnessed OHCA of predominantly cardiac origin to assess an impact of this combined “hyperinvasive approach” on 6 months survival with favourable neurological outcome (primary endpoint), 30 day neurological and cardiac recovery (secondary outcomes), quality of life, safety and cost-effectiveness (tertiary outcomes).

### Hypotheses

We hypothesize, that the combination of above methods will provide increased occurrence of primary and secondary outcomes and will offer a reasonable quality of life for survivors (assessed by SF-36 questionnaire). We further suppose, that the combination of above methods will be cost-effective as assessed by QALY (quality adjusted life year) determination. We also expect same occurrence of complications by using mechanical chest compression device in comparison to manual massage and increased rate of bleeding complications in ECLS, however, compensated by survival benefit in otherwise futile conditions.

### Proposed study protocol

**Figure 1:**
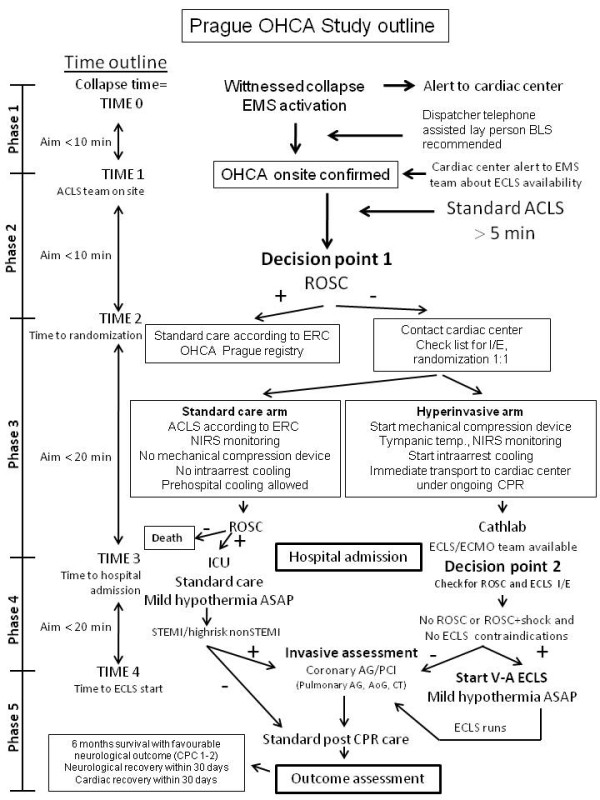
**Prague OHCA study outline.** Abbreviations: ACLS: advanced cardiac life support; AG: angiography; ASAP: as soon as possible; BLS: basic life support; CPC: cerebral performance category; CPR: cardiopulmonary resuscitation; CT: computed tomography; ECLS: extracorporeal life support; EMS: emergency medical service; ERC: European Resuscitation Council; ICU: intensive care unit; I/E: inclusion/exclusion; NIRS: near infrared spectroscopy; OHCA: out of hospital cardiac arrest; ROSC: return of spontaneous circulation; STEMI: ST elevation acute myocardial infarction; TTE: transthoracic echocardiography.

Until stated otherwise, study will be realized only during working hours, ie, 8 AM to 4 PM, to facilitate inhospital logistics and assure presence of key cathlab and ECMO team members. After the official initiation of the study, study coordinator in cardiac center will be notified by a SMS (Short Message Service) alert on every occasion when Prague EMS dispatch center will activate Rapid Response Vehicle (RRV) for witnessed collapse suspected from cardiac arrest or cardiac arrest witnessed by EMS personnel. Coordinator will check for intensive care bed and ECLS capacity and via the dispatch center will notify the EMS team. See the outline of the study (Figure 1) and study phases summarized in Table 1.

**Table 1:** Summary of study phases as per the proposed timeline and expected activities during respective time intervals

**Study phases**	**Activities to be performed**	
***Prerandomization***		
Phase 1 (Time 0 to Time 1)	EMS is activated	
Aimed to be < 10 min	RRV and ambulance car are dispatched	
Time 0 = collapse time	Telephone assisted lay person BLS is started	
Time 1 = EMS team on site	Cardiac center is alerted	
	OHCA confirmed	
Phase 2 (Time 1 to Time 2)	ACLS is started by the first crew on site	
Time 2 = randomization	All initial procedures are performed (defibrillation/s, airway management, i.v. access establishment, etc.)	
	After a minimum of 5 minutes of ACLS guided by EMS physician eligibility for the study is considered (Decision point 1)	
	Randomization is performed by phone call with cardiac center coordinator	
***Postrandomization***	**Standard arm**	**Hyperinvasive arm**
Phase 3 (Time 2 to Time 3)	Continue ACLS according to recent ERC guidelines, start NIRS monitoring, no mechanical compression device used, no intraarrest cooling	Start mechanical compression device, take tympanic temperature, start NIRS monitoring, start intraarrest cooling
Prehospital randomized phase		
Time 3 = hospital admission	ROSC assessment	Immediate transport to cardiac center cathlab under ongoing CPR, continue ACLS according to recent ERC guidelines
	If ROSC, transport to cardiac center ICU	
	Prehospital cooling in case of stable ROSC is allowed	If ROSC during transport, continue transport to cathlab, continue cooling and proceed with invasive assessment
	If death on scene, autopsy at Inst. for Forensic Medicine	If death on scene or during transport, autopsy at Inst. for Forensic Medicine
Phase 4 (Time 3 to Time 4)	Standard post cardiac arrest care, mild hypothermia to 33-34°C ASAP	ROSC and shock assessment, urgent brief TTE
Time 4 = ECLS start – applies for hyperinvasive arm, in standard arm Time 4 = initial assessment	Initial assessment - if STEMI/high risk nonSTEMI proceed to cathlab	ECLS I/E assessment
	Continue NIRS	If no ROSC, or ROSC + shock and no ECLS I/E contraindications – immediate ECLS implantation
	If death, autopsy at Inst. for Forensic Medicine	Immediate invasive assessment (coronary AG, if normal – pulmonary AG, if normal - aortography, eventually brain CT)
		Continue NIRS
		Continue mild hypothermia to 33-34 C
		If death, autopsy at Inst. for Forensic Medicine
Phase 5 (Time 4 to Time 5)	Standard post cardiac arrest care	Continue ECLS until weaning and discontinuation
Time 5 = 6 months evaluation or time of death	Evaluation of cardiac and neurological recovery within 30 days/until discharge	Assess ECLS related adverse events (bleeding, need for blood products)
	6 months survival with CPC 1–2 assessment	Standard post cardiac arrest care
	If death, autopsy at Inst. for Forensic Medicine	Evaluation of cardiac and neurological recovery within 30 days/until discharge
		6 months survival with CPC 1–2 assessment
		If death, autopsy at Inst. for Forensic Medicine

*Abbreviations: ACLS*: advanced cardiac life support; *AG*: angiography; *ASAP*: as soon as possible; *BLS*: basic life support; *CPC*: cerebral performance category; *CPR*: cardiopulmonary resuscitation; *CT*: computed tomography; *ECLS*: extracorporeal life support; *EMS*: emergency medical service; *ICU*: intensive care unit; *I/E*: inclusion/exclusion; *NIRS*: near infrared spectroscopy; *OHCA*: out of hospital cardiac arrest; *ROSC*: return of spontaneous circulation; *STEMI*: ST elevation acute myocardial infarction; *TTE*: transthoracic echocardiography.

On arrival to the scene, patients will be evaluated by an EMS physician to confirm OHCA and standard ACLS (advanced cardiac life support) will be initiated. After a minimum of 5 minutes of ACLS guided by emergency physician and performance of all necessary initial procedures according to recent guidelines and as per physician decision on the scene (ie, defibrillations, airway management, intravenous access establishment) and while the patient is being resuscitated by other EMS team members for continuing cardiac arrest (i.e. no ROSC occurrence, continuous unconsciousness) screening for study eligibility will be performed, see Table 2 for inclusion and exclusion criteria. After the emergency physician on scene evaluates the eligibility criteria and identifies a possibly eligible patient, he directly contacts the cardiac center coordinator by a mobile phone and when consensus on eligibility is established including the bed capacity and ECLS team availability, (Decision point 1 in the project outline), randomization procedure will be performed by a cardiac center coordinator on-line using a computer web based randomization system. Study number will be assigned to the patient and the treatment arm assignment will be notified to the emergency physician on hold. Patients will be randomized in a 1:1 design to hyperinvasive or standard arm.

**Table 2:** Prague OHCA study inclusion and exclusion criteria

** Inclusion criteria**	** Exclusion criteria**
Age ≥18 and ≤ 65 years	OHCA of presumed non-cardiac cause
Wittnessed OHCA of presumed cardiac cause	Unwitnessed collapse
Minimum of 5 minutes of ACLS performed by emergency medical service team without sustained ROSC	Suspected or confirmed pregnancy
Unconsciousness^1^	ROSC within 5 minutes of ACLS performed by EMS team
ECLS team and ICU bed capacity in cardiac center available	Conscious patient
	Known bleeding diathesis or suspected or confirmed acute or recent intracranial bleeding
	Suspected or confirmed acute stroke
	Known severe chronic organ dysfunction or other limitations in therapy
	“Do not resuscitate” order or other circumstances making 180 day survival unlikely
	Known pre-arrest cerebral performance category CPC ≥ 3

*Abbreviations: OHCA*: out-of hospital cardiac arrest; *ACLS*: advanced cardiac life support; *ROSC*: return of spontaneous circulation; *ECLS*: extracorporeal life support; *ICU*: intensive care unit; *EMS*: emergency medical service; *CPC*: cerebral performance category.

^1^ defined as no response to verbal or painful stimuli during ACLS.

In hyperinvasive arm a mechanical chest compression device (LUCAS) will be immediately instituted on scene. Tympanic temperature will be measured, NIRS (near infrared spectroscopy) monitoring and cooling by RhinoChill device will be initiated as soon as possible, realistically immediately after delivering the patient to the ambulance car. Thereafter, patients will be transferred directly to cardiac center cathlab under continuous CPR to fulfill the timeline of reaching ECLS team within 60 minutes after collapse. The use of drugs, further defibrillations or other interventions during transport will follow recent ERC guidelines. On admission to cathlab, overall status, ROSC presence and ECLS inclusion/exclusion criteria will be evaluated (Decision point 2 in the project outline, Figure 1). ECLS eligibility (Table 3): no ROSC or ROSC with ongoing shock state (defined as sustained hypotension below 90 mmHg of systolic pressure or need for bolus doses of vasopressors to maintain the circulation), admission to cathlab not later than 60 minutes after the collapse/initial call to EMS, no signs of death or irreversible organ damage and no contraindications to ECLS institution (known bleeding diathesis, inadequate arterial and venous access for femoro-femoral veno-arterial ECLS). If the ECLS team members reach consensus on ECLS eligibility, it will be started as soon as possible by a standard percutaneous femoro-femoral approach. After ECLS institution, mild hypothermia will be continued by means of extracorporeal circuit cooling and immediate coronary angiography +/− PCI (eventually pulmonary angiography, aortography or head CT if cause of arrest still not obvious) will be performed in all patients. If the patient randomized to hyperinvasive arm reaches ROSC during the transport or after admission to cathlab before ECLS institution, he will undergo initial clinical assessment, ECG, urgent echocardiography and will continue with invasive investigations as mentioned above.

**Table 3:** Inclusion and exclusion criteria for initiation of ECLS in Prague OHCA study protocol

**Inclusion criteria**	**Exclusion criteria**
No ROSC or ROSC with ongoing shock (defined as sustained hypotension below 90 mmHg of systolic pressure or need for bolus doses of vasopressors to maintain the circulation)	Signs of death or irreversible organ damage
Admission to cathlab not later than 60 minutes after the collapse/initial call to EMS^1^	Known bleeding diathesis
Consensus of ECMO team members on ECLS initiation	Inadequate arterial and/or venous access for femoro-femoral cannulation

*Abbbreviations: ECMO*: extracorporeal membrane oxygenation; *ECLS*: extracorporeal life support; *ROSC*: return of spontaneous circulation.

^1^if collapse time is not exactly known, initial call to EMS will be considered.

Patients randomized to a standard arm will be managed by continued ACLS on site. The use of drugs, further defibrillations or other interventions will follow recent ERC guidelines. Patients will also undergo NIRS monitoring. If ROSC is achieved, patients will be transferred to the same hospital to one of intensive care units (ICU), emergency room or cathlab observational area and coronary angiography/PCI will be performed in all patients with STEMI (ST elevation myocardial infarction) identified on any post ROSC 12 lead ECG or high risk nonSTEMI defined by persistent ST segment depressions or high risk for in hospital death according to GRACE risk score, ie GRACE risk score > 140 points, predicted mortality > 3%, http://www.outcomes.org/grace) [45,46]. Mild therapeutic hypothermia will be started as soon as possible after ROSC (including prehospital cooling on a discretion of the emergency physician), intraarrest cooling will not be allowed in the standard arm. Stopping resuscitation efforts will follow the recent ERC guidelines which state continuing CPR while VF/VT is present and at least 20 minutes when asystoly is an initial rhythm, however, definitive decision will be on a discretion of EMS physician.

### Randomization process

The online randomization process during the ongoing CPR has been selected to overcome selection bias in cluster randomizations, because study arm assignment before starting CPR can influence the decision making [24]. Accordingly, chest compression device, i.e. LUCAS has to be carried to all putative OHCA victims and will be used only when randomization to hyperinvasive arm occurs. This is somewhat inconvenient to EMS crew, however, necessary to avoid unintentional bias. In contrary to this, intranasal cooling will be started in the ambulance vehicle, because carrying another device to the scene would be too demanding and time delay for transporting a patient from the scene to an ambulance car will be negligible. The randomization phone call between the emergency physician and coordinating cardiologist/intensivist at the cardiac center is a crucial activity to properly enroll the patients and fulfill the inclusion/exclusion criteria. These phone calls have been already trained during the seminars and investigator meetings and should not last more than 60 sec. At the time of the phone call, all the vital procedures performed by the EMS physician are already done, and at least 3 other rescue persons are on the scene. Thus, the physician can safely make this phone call, while others are continuing the CPR. The web based randomization system has been chosen, to maximally shorten the necessary time. Only following information will be requested after logging into the system: patient estimated age and gender and confirmation of I/E criteria. Immediately thereafter the patient number and treatment assignment will be generated. For the case of web randomization system failure, envelopes with treatment arm assignment will be prepared in the coordinating center, just next to the computer used for randomization.

### Post resuscitation care

All patients admitted to hospital in both arms will have immediate biochemical evaluation, continuing neurological monitoring by near-infrared spectroscopy and brief urgent echocardiography. Nasal cooling in hyperinvasive arm will continue until transition to systemic cooling either by ECLS or by intravascular cooling catheter or standard surface cooling combined with rapid intravenous administration of cold normal saline (4°C, 20–30 ml/kg/hour according to hemodynamic status) to reach target temperature of 33°C as soon as possible. Target core temperature will be maintained for 24 hours. After the maintenance period, core temperature will be slowly raised to normothermia of 37°C during 8 hours with a rewarming rate of 0.5°C/hour in both groups. Body temperature will then be maintained at normothermia 37 ±0.5°C until 72 hours from sustained ROSC in both treatment groups, as long as the patient is in the ICU, using pharmacotherapy or other temperature management systems whenever applicable. EMS and hospital personnel will not be blinded during the treatment. All other post arrest critical care management will follow recent ERC guidelines and other generally accepted approaches [3,6,7,26].

Since the official initiation of the study, all patients resuscitated by Prague EMS not fulfilling eligibility criteria for this study will also be followed for outcome assessment and will constitute the third comparative group, “Prague OHCA study registry” patients (see the outline of the study).

### Devices used

LUCAS (Lund University Cardiac Arrest System, Physio-Control Inc./Jolife AB, Lund, Sweden) device for mechanical chest compressions, http://www.physio-control.com/LUCAS.

RhinoChill device (BeneChill, Inc., San Diego, Calif, USA) device for intraarrest intranasal evaporative cooling, http://www.benechill.com/wp/rhinochill-trade/rhinochill-device.

For ECLS, MAQUET PLS console (MAQUET Cardiopulmonary AG, Hirrlingen, Germany) and Rotaflow RF 32 centrifugal pump with Quadrox PLS hollow fibre BIOLINE® coated membrane oxygenator (MAQUET Cardiopulmonary AG, Hirrlingen, Germany) or alternatively MAQUET custom made tubing set and Eurosets ECMO oxygenator A.L.ONE (EUROSETS s.r.l., Medolla, Italy) and a mechanical gas blender (Sechrist, Anaheim, CA, USA) will be used. Edwards cannulae (Fem-Flex Cannulae, Edwards Lifesciences Research Medical Inc., Midvale, UT, USA) or alternatively MAGUET cannulae will be used for femoro-femoral cannulation. Surgical standby will be available to assist in case of any cannulation difficulties. Ultrasound guided antegrade perfusion cannula will be implanted whenever limb ischemia is being detected clinically or by regional oxygen saturation decrease (see below). Patients on ECLS will be continuously anticoagulated with heparin until contraindicated, targeted to ACT (activated clotting time) of 180–220 sec or aPTT of 50–80 sec.

For regional oxygen saturation (neuromonitoring and lower extremities´ perfusion), an INVOS device (INVOS Cerebral/Somatic Oximeter, Covidien, Boulder, CO, USA) will be used for near infrared spectroscopy measurement during both prehospital and inhospital phase. During prehospital phase, only brain regional saturations will be measured. During inhospital phase, in standard arm, brain saturations will be further measured, in hyperinvasive arm, 4 channels will be used to measure both hemispheres and also lower extremities´ saturations for early detection and further monitoring of possible lower limb ischemia due to ECMO cannula femoral artery obstruction.

### Ethics, safety and registration

The study has been approved by the Institutional Review Board of the General University Hospital and First Faculty of Medicine, Charles University in Prague. Ethical considerations for treating subjects without their expressed consent are in accordance with the Helsinki Declaration of 1964, revised in 2008. The subject’s legal representative will be informed of the subject’s study participation as soon as practical, and patients who regain normal neurological function will be asked to provide their consent for use of the data. The study has been registered under ClinicalTrials.gov identifier: NCT01511666.

### Data safety monitoring board (DSMB) and data monitoring

An independent DSMB consisting of experts in the field of cardiac arrest will follow the overall study progression and integrity. DSMB will meet after inclusion of every 30 patients or every 6 months, whatever comes first, to evaluate the progress in the study and review all adverse events. Study data will be monitored by a professional contracted CRO (contract research organization).

### Outcomes

#### Primary outcome

Composite endpoint of 6 months survival with good neurological outcome (CPC 1–2).

### Secondary outcomes

1/30 day neurological recovery - defined as no or minimal neurological impairment (CPC 1 or 2) at any timepoint within first 30 days after initial cardiac arrest. Neurological status will be evaluated by determining CPC value every day on ICU and on ICU discharge, on hospital discharge and/or on day 7, 14 ± 2 days, 30 ± 2 days, 90 ± 3 days and 180 ± 3 days. Neurological assessment on hospital discharge will be provided by a neurologist blinded to study protocol and the treatment assignment. Neurological status on ECLS will follow the same timeline as stated above, brain death determination will respect the valid laws of the country.

2/30 day cardiac recovery - defined as no need for pharmacological or mechanical cardiac support. Cardiac status will be evaluated every day on ICU and on ICU discharge. Systolic function will be measured by echocardiography on day 1 on ICU, before hospital discharge and on day 180 ± 3 days.

### Tertiary outcomes

Early outcome will also be monitored by means of ROSC achievement, defined as a palpable puls and measurable blood pressure without ECLS and ROSB (return of spontaneous beating) on ECLS, defined as palpable pulse or pulsatile flow on arterial invasive blood pressure curve. ECLS weaning will be considered when cardiac function starts to improve, meaning that spontaneous pulsatile flow will be achieved and stable or decreasing doses of inotropes and vasopressors will be used for more than 24 hours. Stable circulation for more than 24 hours after stopping ECLS will be considered to be a weaning success. In case of failed ECLS weaning due to a persistent heart dysfunction and good neurological and overall recovery, patients will be considered for ventricle assist device (VAD) implantation or heart transplantation. In nonweanable ECLS situation and poor neurological outcome, the patient will be considered to become a nonbeating heart donor. For patients not suitable for VAD/heart transplantation nor for nonbeating heart donorship consideration, ECLS will be withdrawn. All survivors will be followed until discharge home or to a long term care or rehabilitation center and thereafter in an Outpatient Heart Failure Clinic of the coordinating center. Quality of life will be assessed using SF-36 questionnaire on discharge and during the 6 months visit. Safety of the invasive methods will be monitored by adverse events occurrence in survivors and organ damage will be assessed on autopsies in nonsurvivors. Cost-effectiveness will be evaluated by determination of QALY (Quality Adjusted Life Year). Patients who die, will undergo autopsy at the Department of Forensic Medicine and Toxicology, General University Hospital and First Faculty of Medicine.

#### Timeline

During the initial months of 2012 we expect a development of web based randomization and database system including CRF (case report form). EMS personnel has been trained in all necessary procedures and methods (i.e. LUCAS and RhinoChill device) during 2011 (3 seminars per 4 hours) and routinely uses LUCAS device in cardiac arrest setting. A simulation study is planned for the first half of 2012, i.e. 3–5 patients will be “randomized” to hyperinvasive arm, to be sure, that the protocol is feasible, all procedures are well trained and ECLS team is able to meet quickly and connect the patient to ECLS as per scheduled outline. Only thereafter and following DSMB recommendation a real randomized study phase will be initiated. We expect approximately 40 patients to be enrolled yearly until planned number of patients according to power analysis, or DSMB stops the study.

### Statistical considerations

Initial statistical analysis was performed taking into account three proposed groups of patients. First, patients who will not be randomized, i.e. Prague OHCA study registry patients (see study outline on Figure 1). These patients will not fulfill inclusion/exclusion criteria mainly by means of not having “refractory” cardiac arrest, ie, successful ROSC will be reached within 5–10 minutes of ACLS provided by EMS physician staffed team. According to Prague EMS study assessing overall outcome of all CPRs in Prague in 2008 [4] with 15% overall short term survival with favourable neurological outcome (discharged home), we expect better, approximately 20–30% of “primary outcome” occurrence in this comparative group of patients. The other two groups in randomized part of the study will yield standard and hyperinvasive arm patients with rather worse outcomes. We expect 90% mortality in standard arm, or alternatively stated, 10% six-month survival with favourable neurological outcome. The characteristics of patients and their procedures not included in randomization process will be tracked and treated as possible confounding factors and included in statistical analysis as cofactors when necessary.

### The power analysis of the study

The power analysis was computed for superiority of hyperinvasive approach over standard approach, i.e. using two tailed test with the α = 0.05 and desired power 0.9. In the standard arm 10% six-month survival with favourable neurological outcome (primary outcome) is assumed and 15% increase in primary outcome occurrence (6 month survival with favourable neurological outcome) is considered as clinically relevant. Three scenarios with 10%, 15% and 20% increase of primary outcome were computed. The analysis was computed using ADDPLAN BASE version 6.0 (Aptiv Solutions, Cologne, Germany, 2011).

Scenario 1: standard (10%) vs. hyperinvasive (20%) groups with allocation ratio 1; two tailed test with α = 0.05 and power = 0.9.

A design with a maximum of K = 4 stages was chosen. The critical values and the test characteristics of the group sequential test design were calculated for a Pampallona and Tsiatis design with boundary shape parameter Delta0 = 0.00 to reject H0, and boundary shape parameter Delta1 = 0.00 to reject H1.

**Figure 2:**
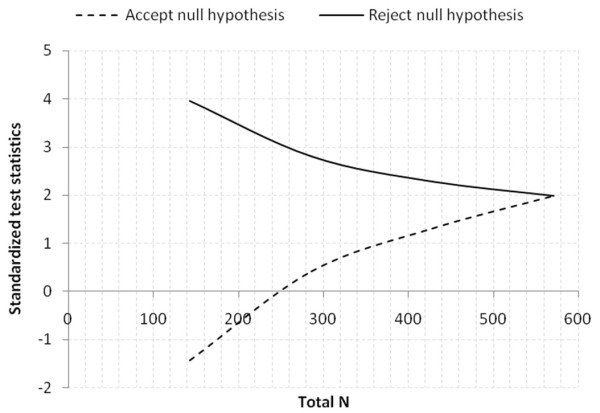
Graphical delineation for scenario 1, estimated 10% increase of primary outcome in hyperinvasive (20%) vs. standard (10%) groups.

This yields a total of 285.7 + 285.7 = 571.4 observations. For comparison, the sample size in a fixed sample size design is n1 = 265.9, n2 = 265.9. The expected (average) total sample size under the alternative hypothesis is 404.1, under a value midway between H0 and H1 it is 444.3, and under the null hypothesis it is 403.1, see Figure 2.

Scenario 2: standard (10%) vs. hyperinvasive (25%) groups with allocation ratio 1; two tailed test with α = 0.05 and power = 0.9.

A design with a maximum of K = 4 stages was chosen. The critical values and the test characteristics of the group sequential test design were calculated for a Pampallona and Tsiatis design with boundary shape parameter Delta0 = 0.00 to reject H0, and boundary shape parameter Delta1 = 0.00 to reject H1.

**Figure 3:**
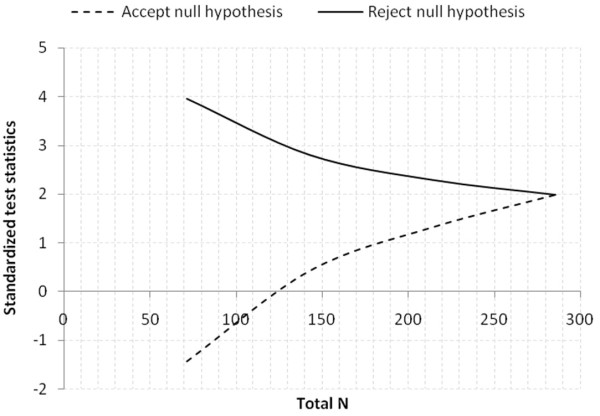
Graphical delineation for scenario 2, estimated 15% increase of primary outcome in hyperinvasive (25%) vs. standard (10%) groups.

This yields a total of 142.7 + 142.7 = 285.4 observations. For comparison, the sample size in a fixed sample size design is n1 = 132.8, n2 = 132.8. The expected (average) total sample size under the alternative hypothesis is 201.8, under a value midway between H0 and H1 it is 221.9, and under the null hypothesis it is 201.3, see Figure 3.

Scenario 3: standard (10%) vs. hyperinvasive (30%) groups with allocation ratio 1; two tailed test with α = 0.05 and power = 0.9.

A design with a maximum of K = 4 stages was chosen. The critical values and the test characteristics of the group sequential test design were calculated for a Pampallona and Tsiatis design with boundary shape parameter Delta0 = 0.00 to reject H0, and boundary shape parameter Delta1 = 0.00 to reject H1.

**Figure 4:**
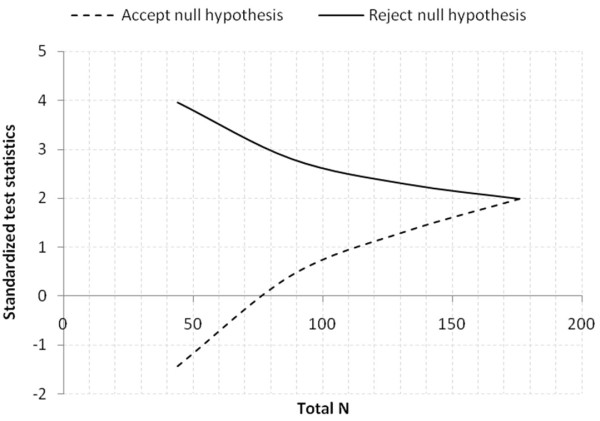
Graphical delineation for scenario 3, estimated 20% increase of primary outcome in hyperinvasive (30%) vs. standard (10%) groups.

This yields a total of 88.1 + 88.1 = 176.2 observations. For comparison, the sample size in a fixed sample size design is n1 = 82.0, n2 = 82.0. The expected (average) total sample size under the alternative hypothesis is 124.6, under a value midway between H0 and H1 it is 137.0, and under the null hypothesis it is 124.3002, see Figure 4.

### Cooperation

The project will be executed in a close cooperation of Complex Cardiac Center of General University Hospital with Prague Emergency Medical Service. Both institutions cooperate on a day by day basis during the routine care for cardiac arrest patients including admissions during ongoing CPR. In these occasions the cardiac center is alerted early and the catheterization and ECLS team is prepared at the cathlab. The decision on ECLS initiation is always reached consensually within the ECMO team members [47].

### Readiness of cooperating institutions

Complex Cardiac Center of General Teaching Hospital, Charles University in Prague admits approximately 100 patients after cardiac arrest yearly. Approximately 20 patients per year are treated by ECLS under ECMO team guidance, coordinated by principal investigator of this project (JB). Until now, 67 patients have been treated by ECMO and both clinical [47-50] and experimental [51] results and experiences have been published. Cardiac center is located within the city center.

Prague EMS provides a prehospital urgent care within the capitol Prague by a rendezvous system with rapid response vehicles (RRV) staffed by emergency physicians and ambulance cars staffed by paramedics and intensive care nurses. Necessary devices, i.e. LUCAS for mechanical chest compressions and RhinoChill for intranasal evaporative cooling are currently available for all RRVs. An INVOS device (INVOS Cerebral/Somatic Oximeter, Covidien, Boulder, CO, USA) for NIRS monitoring is also available, however only for one inspector car. This car is alerted routinely in every resuscitated OHCA in Prague for CPR assistance, however, inclusion into the study is possible without INVOS availability. An analysis of cardiac arrest occurrence in Prague in 2007–2010 confirms a frequent occurrence in the center of the city, which is a favourable precondition to reach short transport times to cardiac center (personal communication with OF – data not published).

## Discussion

This complex and logistically demanding project has been designed to collect a clear result stating whether the combination of modern sophisticated methods improves or not the unfavourable prognosis of cardiac arrest patients. The project differs from other already performed studies by randomizing the patients to a combination of potentially beneficial methods used in cardiac arrest. Such a combination or “hyperinvasive” approach has not been performed so far, as per our knowledge. The underlying “all in one” concept is to maximize the beneficial effect on outcome of cardiac arrest patients, i.e. to keep the end-organ perfusion by mechanical chest compression, to avoid neurological damage by early intraarrest intranasal evaporative cooling and to bridge to ECLS with further invasive evaluation to identify and immediately treat the cause of refractory arrest by means of percutaneous techniques, if cause is identified. Of course, we may also expect untreatable causes of sudden refractory arrest like aortic aneurysmal rupture, intracranial bleeding with occipital conus, unidentified trauma with severe inner organ damage, initially unrecognizable poisoning etc. However, we also expect a significant proportion of potentially treatable causes, mainly the ongoing ischemia due to acute coronary obstruction and massive pulmonary embolism with severe right ventricle failure. As per available data [3] and our own experience (Smid, Belohlavek – data not published yet), in 80% of OHCA victims, cardiac etiology can be identified with diagnostic accuracy in prehospital phase of approximately 75% [52]. Two thirds of these patients suffer either acute coronary syndrome or pulmonary embolism. In remaining one third of patients, complications of chronic heart failure is the the most frequent cause.

A key prerequisite for successful result is strict compliance with proposed timeline (see the outline of the study on Figure 1) and adequate use of all devices. Therefore, study preparation phase lasted over one year. All RRV crews had to become perfectly familiar with LUCAS device and were repeatedly trained in application of this device. The same applies for prehospital RhinoChill device use and also for an acute implantantion of ECLS by ECMO team in cathlab. All study investigators, cathlab and ICU personnel have also been repeatedly trained in study protocol. Moreover, initially we plan at least 3–5 patients to be “randomized” to hyper invasive approach (simulation phase) before real randomized study starts, to prove the concept and feasibility of the protocol. This allows us to recognize potential logistic barriers or any other misconceptions. Further on, the pilot phase of the study will be performed only within working hours, ie, 8 AM to 4 PM and only when principal investigator is present, to optimize for personal and organizational demands. Based on initial result and feasibility of the whole concept, after randomization of 30 patients, DSMB will decide whether to continue the study or not.

We also seriously considered the definition of “refractory” cardiac arrest, as this definition varies in available studies [31,33]. We expect the average time to randomization in our proposed study to be around 20 minutes, considering following time intervals: 9 minutes is an average response time for a RRV to reach the patient with OHCA in Prague [4]; a minimum of 5 minutes of ACLS by the EMS team on scene including performance of all necessary procedures (defibrillation or defibrillations, airway management, intravenous access establishment), we actually expect this interval to last longer, ie, approximately 10 minutes and 1–2 minutes of randomization phone call with cardiac center coordinator.

The protocol is opened for sharing by other cardiac centers with available ECLS and cathlab teams trained to admit patients with refractory cardiac arrest under ongoing CPR. A prove of concept study will be started soon. The aim of the authors is to establish a net of centers for a multicenter trial initiation in future.

### Contribution of the project and clinical consequences

Potential contribution is crucial taking into account the socio-economic consequences of cardiac arrest. Cardiac arrest often affects relatively young fully active persons and portends high mortality mainly due to severe neurological damage causing both personal tragedies to patients and to their relatives and increases in health care costs. If the beneficial effect of proposed combination of therapeutical methods were proved, it might have a profound influence on logistics of emergency care for cardiac arrest patients, mainly in cities and urban agglomerations similar to Prague, i.e. in cities with well organized prehospital care, short arrival times and within city center located cardiac center with emergently available ECLS and cathlab team capacity.

## Conclusion

Authors introduce and offer a protocol of a proposed randomized study enrolling patients with witnessed OHCA presumably of cardiac origin planned to be initiated in Prague in 2012. Study will compare hyperinvasive approach encompassing prehospital intraarrest cooling, mechanical chest compression, veno-arterial ECLS and immediate invasive diagnostics in all patients compared to a standard of care. The protocol is opened for sharing by other cardiac centers with readily available ECLS and cathlab teams used to cooperate with emergency medical services to admit patients with refractory cardiac arrest under ongoing CPR to establish a net of centers for a multicenter trial realization in future.

## Abbreviations

ACLS: Advanced cardiac life support; AG: Angiography; AoG: Aortography; ASAP: As soon as possible; BLS: Basic life support; CA: Cardiac arrest; CPC: Cerebral performance category; CPR: Cardiopulmonary resuscitation; CRF: Case report form; CT: Computed tomography; DSMB: Data safety monitoring board; ECMO: Extracorporeal membrane oxygenation; ECLS: Extracorporeal life support; EMS: Emergency medical service; ICU: Intensive care unit; I/E: Inclusion/exclusion; IHCA: In hospital cardiac arrest; LUCAS: Lund University Cardiac Arrest System; NIRS: Near infrared spectroscopy; OHCA: Out of hospital cardiac arrest; PCI: Percutaneous coronary intervention; QALY: Quality Adjusted Life Year; RRV: Rapid response vehicle; ROSC: Return of spontaneous circulation; STEMI: ST elevation acute myocardial infarction; TTE: Transthoracic echocardiography; V-A: Veno-arterial.

## Competing interests

A Puroklima a.s. company, a distributor for Czech Republic for Benechill provided eight Rhinochill devices and for the purpose of the study will also provide the application sets and evaporative liquid per substantially reduced cost. Likewise, a Physio-Control, division of Medtronic Czechia s.r.o. company provided eight LUCAS devices for the purpose of the study. Both device will be provided to Prague EMS to equip all RRVs. Maquet company provided the MAQUET PLS device for ECLS. Covidien company provided the INVOS devices for NIRS measurement. The main author (JB) has received two lecture honoraria from Maquet company, Czech Republic.

## Authors’ contribution

JB is a main author, concepted and designed the study and prepared the manuscript. JJ and VK prepared the statistical power analysis, will assist in data management. KK, OF, MP, JD, RS, JV and ZS substantially contributed to conception and design and will be responsible for acquisition, verification and interpretation of prehospital data. OS, JH, MB and AL participated on conception and design and will be responsible for acquisition, verification and interpretation of inhospital data. VM and AL obtained research funding and AL has also given a final approval of the version to be published. All authors read and approved the final manuscript.

## References

[B1] Goldberger JJ Cain ME Hohnloser SH Kadish AH Knight BP Lauer MS Maron BJ Page RL Passman RS Siscovick D Stevenson WG Zipes DP American Heart Association Council on Clinical Cardiology; American Heart Association Council on Epidemiology and Prevention; American College of Cardiology Foundation; Heart Rhythm Society. American Heart Association/american College of Cardiology Foundation/heart Rhythm Society scientific statement on noninvasive risk stratification techniques for identifying patients at risk for sudden cardiac death: a scientific statement from the American Heart Association Council on Clinical Cardiology Committee on Electrocardiography and Arrhythmias and Council on Epidemiology and Prevention Heart Rhythm 2008 5 e1 e21 18929319 10.1016/j.hrthm.2008.05.031

[B2] Zheng ZJ Croft JB Giles WH Mensah GA Sudden cardiac death in the United States, 1989 to 1998 Circulation 2001 104 2158 2163 10.1161/hc4301.098254 11684624

[B3] Hazinski MF Nolan JP Billi JE Böttiger BW Bossaert L de Caen AR Deakin CD Drajer S Eigel B Hickey RW Jacobs I Kleinman ME Kloeck W Koster RW Lim SH Mancini ME Montgomery WH Morley PT Morrison LJ Nadkarni VM O’Connor RE Okada K Perlman JM Sayre MR Shuster M Soar J Sunde K Travers AH Wyllie J Zideman D Part 1: Executive summary: 2010 International Consensus on Cardiopulmonary Resuscitation and Emergency Cardiovascular Care Science With Treatment Recommendations Resuscitation 2010 81 Suppl 1 e1 e25 20956042 10.1016/j.resuscitation.2010.08.002 PMC7115798

[B4] Franěk O Pokorná M Sukupová P Pre-hospital cardiac arrest in Prague, Czech Republic—The Utstein-style report Resuscitation 2010 81 831 835 10.1016/j.resuscitation.2010.03.005 20413205

[B5] Kirves H Skrifvars MB Vahakuopus M Ekstrom K Martikainen M Castren M Adherence to resuscitation guidelines during prehospital care of cardiac arrest patients Eur J Emerg Med 2007 14 75 81 10.1097/MEJ.0b013e328013f88c 17496680

[B6] Sunde K Pytte M Jacobsen D Mangschau A Jensen LP Smedsrud C Draegni T Steen PA Implementation of a standardised treatment protocol for post resuscitation care after out-of-hospital cardiac arrest Resuscitation 2007 73 29 39 10.1016/j.resuscitation.2006.08.016 17258378

[B7] Gaieski DF Band RA Abella BS Neumar RW Fuchs BD Kolansky DM Merchant RM Carr BG Becker LB Maguire C Klair A Hylton J Goyal M Early goal-directed hemodynamic optimization combined with therapeutic hypothermia in comatose survivors of out-of-hospital cardiac arrest Resuscitation 2009 80 418 424 10.1016/j.resuscitation.2008.12.015 19217200

[B8] Deem S Hurford WE Respiratory controversies in the critical care setting. Should all patients be treated with hypothermia following cardiac arrest? Respir Care 2007 52 443 450 17417978

[B9] Wijdicks EF Hijdra A Young GB Bassetti CL Wiebe S Practice parameter: prediction of outcome in comatose survivors after cardiopulmonary resuscitation (an evidence-based review): report of the Quality Standards Subcommittee of the American Academy of Neurology Neurology 2006 67 203 210 10.1212/01.wnl.0000227183.21314.cd 16864809

[B10] Rossetti AO Oddo M Logroscino G Kaplan PW Prognostication after cardiac arrest and hypothermia: a prospective study Ann Neurol 2010 67 301 307 20373341 10.1002/ana.21984

[B11] Nielsen N Friberg H Gluud C Herlitz J Wetterslev J Hypothermia after cardiac arrest shloud be further evaluated – A systematic review of randomised trials with meta-analysis and trial sequential analysis Int J Cardiol 2011 15 3 333 341 20591514 10.1016/j.ijcard.2010.06.008

[B12] Nielsen N Sunde K Hovdenes J Riker RR Rubertsson S Stammet P Nilsson F Friberg H Hypothermia Network. Adverse events and their relation to mortality in out-of-hospital cardiac arrest patients treated with therapeutic hypothermia Crit Care Med 2011 39 57 64 10.1097/CCM.0b013e3181fa4301 20959789

[B13] Nielsen N Hovdenes J Nilsson F Rubertsson S Stammet P Sunde K Valsson F Wanscher M Friberg H Hypothermia Network. Outcome, timing and adverse events in therapeutic hypothermia after out-of-hospital cardiac arrest Acta Aneasthesiol Scand 2009 53 926 934 10.1111/j.1399-6576.2009.02021.x 19549271

[B14] Bělohlávek J Šmíd O Therapeutic hypothermia after cardiac arrest: why and for how long? Vnitr Lek 2011 57 23 26 21351658

[B15] Pfeifer R Jung C Purle S Lauten A Yilmaz A Surber R Ferrari M Figulla HR Survival does not improve when therapeutic hypothermia is added to post-cardiac arrest care Resuscitation 2011 82 1168 1173 10.1016/j.resuscitation.2011.05.024 21715080

[B16] Scolletta S Taccone FS Nordberg P Donadello K Vincent JL Castren M Intra-arrest hypothermia during cardiac arrest: a systematic review Crit Care 2012 16 R41 10.1186/cc11235 22397519 PMC3681365

[B17] Garrett JS Studnek JR Blackwell T Vandeventer S Pearson DA Heffner AC Reades R The association between intra-arrest therapeutic hypothermia and return of spontaneous circulation among individuals experiencing out of hospital cardiac arrest Resuscitation 2011 82 21 25 10.1016/j.resuscitation.2010.09.473 21036449

[B18] Riter HG Brooks LA Pretorius AM Ackermann LW Kerber RE Intra- arrest hypothermia: both cold liquid ventilation with perfluorocarbons and cold intravenous saline rapidly achieve hypothermia, but only cold liquid ventilation improves resumption of spontaneous circulation Resuscitation 2009 80 561 566 10.1016/j.resuscitation.2009.01.016 19249149 PMC2706261

[B19] Menegazzi JJ Rittenberger JC Suffoletto BP Logue ES Salcido DD Reynolds JC Sherman LD Effects of pre‑arrest and intra- arrest hypothermia on ventricular fibrillation and resuscitation Resuscitation 2009 80 126 132 10.1016/j.resuscitation.2008.09.002 18952346 PMC2720166

[B20] Castrén M Nordberg P Svensson L Taccone F Vincent JL Desruelles D Eichwede F Mols P Schwab T Vergnion M Storm C Pesenti A Pachl J Guérisse F Elste T Roessler M Fritz H Durnez P Busch HJ Inderbitzen B Barbut D Intra-arrest transnasal evaporative cooling: a randomized, prehospital, multicenter study (PRINCE: Pre-ROSC IntraNasal Cooling Effectiveness) Circulation 2010 17 7 729 736 10.1161/CIRCULATIONAHA.109.931691 20679548

[B21] Krep H Mamier M Breil M Heister U Fischer M Hoeft A Out-of hospital cardiopulmonary resuscitation with the AutoPulse system: a prospective observational study with a new load-distributing band chest compression device Resuscitation 2007 73 86 95 10.1016/j.resuscitation.2006.08.027 17254691

[B22] Hallstrom A Rea TD Sayre MR Christenson J Anton AR Mosesso VN Jr Van Ottingham L Olsufka M Pennington S White LJ Yahn S Husar J Morris MF Cobb LA Manual chest compression vs use of an automated chest compression device during resuscitation following out-of-hospital cardiac arrest: a randomized trial JAMA 2006 295 2620 2628 10.1001/jama.295.22.2620 16772625

[B23] Ong ME Ornato JP Edwards DP Dhindsa HS Best AM Ines CS Hickey S Clark B Williams DC Powell RG Overton JL Peberdy MA Use of an automated, load distributing band chest compression device for out-of-hospital cardiac arrest resuscitation JAMA 2006 295 2629 2637 10.1001/jama.295.22.2629 16772626

[B24] Lerner EB Persse D Souders CM Sterz F Malzer R Lozano M Jr Westfall M Brouwer MA van Grunsven PM Whitehead A Olsen JA Herken UR Wik L Design of the Circulation improving resuscitation care (CIRC) trial: A new state of the art design for out-of hospital cardiac arrest research Resuscitation 2011 82 294 299 10.1016/j.resuscitation.2010.11.013 21196070

[B25] ZOLL Medical Corporation press release CIRC (Circulation improving resuscitation care) trial concludes succesfully 2011 USA: Chelmsford, MASS

[B26] Nolan JP Soar J Zideman DA Biarent D Bossaert LL Deakin C Koster RW Wyllie J Böttiger B ERC Guidelines Writing Group. European Resuscitation Council Guidelines for Resuscitation 2010. Executive summary Resuscitation 2010 81 1219 1276 10.1016/j.resuscitation.2010.08.021 20956052

[B27] Mégarbane B Leprince P Deye N Résière D Guerrier G Rettab S Théodore J Karyo S Gandjbakhch I Baud FJ Emergency feasibility in medical intensive care unit of extracorporeal life support for refractory cardiac arrest Intensive Care Med 2007 33 758 764 10.1007/s00134-007-0568-4 17342517

[B28] Massetti M Tasle M Le Page O Deredec R Babatasi G Buklas D Thuaudet S Charbonneau P Hamon M Grollier G Gerard JL Khayat A Back from irreversibility: extracorporeal life support for prolonged cardiac arrest Ann Thorac Surg 2005 79 178 183 10.1016/j.athoracsur.2004.06.095 15620939

[B29] Chen JS Ko WJ Yu HY Lai LP Huang SC Chi NH Tsai CH Wang SS Lin FY Chen YS Analysis of the outcome for patients experiencing myocardial infarction and cardiopulmonary resuscitation refractory to conventional therapies necessitating extracorporeal life support rescue Crit Care Med 2006 34 950 957 10.1097/01.CCM.0000206103.35460.1F 16484889

[B30] Chen YS Lin JW Yu HY Ko WJ Jerng JS Chang WT Chen WJ Huang SC Chi NH Wang CH Chen LC Tsai PR Wang SS Hwang JJ Lin FY Cardiopulmonary resuscitation with assisted extracorporeal life-support versus conventional cardiopulmonary resuscitation in adults with inhospital cardiac arrest: an observational study and propensity analysis Lancet 2008 372 554 561 10.1016/S0140-6736(08)60958-7 18603291

[B31] Liu Y Cheng YT Chang JC Chao SF Chang BS Extracorporeal membrane oxygenation to support prolonged conventional cardiopulmonary resuscitation in adults with cardiac arrest from acute myocardial infarction at a very low-volume centre Interact Cardiovasc Thorac Surg 2011 12 389 393 10.1510/icvts.2010.256388 21172947

[B32] Morimura N Sakamoto T Nagao K Asai Y Yokota H Tahara Y Atsumi T Nara S Hase M Extracorporeal cardiopulmonary resuscitation for out-of-hospital cardiac arrest: A review of the Japanese literature Resuscitation 2011 82 10 14 10.1016/j.resuscitation.2010.08.032 20934798

[B33] Le Guen M Nicolas-Robin A Carreira S Raux M Leprince P Riou B Langeron O Extracorporeal life support following out-of-hospital refractory cardiac arrest Crit Care 2011 15 R29 10.1186/cc9976 21244674 PMC3222065

[B34] Morris MC Wernovsky G Nadkarni VM Survival outcomes after extracorporeal cardiopulmonary resuscitation instituted during active chest compressions following refractory in-hospital pediatric cardiac arrest Pediatr Crit Care Med 2004 5 440 446 10.1097/01.PCC.0000137356.58150.2E 15329159

[B35] Thiagarajan RR Laussen PC Rycus PT Bartlett RH Bratton SL Extracorporeal Membrane Oxygenation to Aid Cardiopulmonary Resuscitation in Infants and Children Circulation 2007 116 1693 1700 10.1161/CIRCULATIONAHA.106.680678 17893278

[B36] Kane DA Thiagarajan RR Wypij D Scheurer MA Fynn-Thompson F Emani S del Nido PJ Betit P Laussen PC Rapid-response extracorporeal membrane oxygenation to support cardiopulmonary resuscitation in children with cardiac disease Circulation 2010 122 11 Suppl S241 S248 20837920 10.1161/CIRCULATIONAHA.109.928390

[B37] Lebreton G Pozzi M Luyt CE Chastre J Carli P Pavie A Leprince P Vivien B Out-of-hospital extra-corporeal life support implantation during refractory cardiac arrest in a half-marathon runner Resuscitation 2011 82 1239 1242 10.1016/j.resuscitation.2011.04.002 21536365

[B38] Arlt M Philipp A Voelkel S Graf BM Schmid C Hilker M Out-of-hospital extracorporeal life support for cardiac arrest-A case report Resuscitation 2011 82 1243 1245 10.1016/j.resuscitation.2011.03.022 21536364

[B39] Peek GJ Community extracorporeal life support for cardiac arrest - when should it be used? Resuscitation 2011 82 1117 10.1016/j.resuscitation.2011.06.006 21741746

[B40] Haneya A Philipp A Diez C Schopka S Bein T Zimmermann M Lubnow M Luchner A Agha A Hilker M Hirt S Schmid C Müller T A 5-year experience with cardiopulmonary resuscitation using extracorporeal life support in non-postcardiotomy patients with cardiac arrest Resuscitation 2012 20 In press [Epub ahead of print] 10.1016/j.resuscitation.2012.07.009 22819880

[B41] Jaski BE Ortiz B Alla KR Smith SC Jr Glaser D Walsh C Chillcott S Stahovich M Adamson R Dembitsky W A 20-year experience with urgent percutaneous cardiopulmonary bypass for salvage of potential survivors of refractory cardiovascular collapse J Thorac Cardiovasc Surg 2010 139 753 757 10.1016/j.jtcvs.2009.11.018 20176219

[B42] ECC Committee, Subcommittee and Task Force of the American Heart Association 2005 American Heart Association Guidelines for Cardiopulmonary resuscitation and emergency cardiovascular care Circulation 2005 112 suppl IV IV1-203 16314375 10.1161/CIRCULATIONAHA.105.166550

[B43] Frederiksson M Aune S Bang A Thoren AB Lindqvist J Karisson T Herlitz J Cardiac arrest outside and inside hospital in a community: mechanisms behind the differences in outcome and outcome in relation to time of arrest Am Heart J 2010 159 749 756 10.1016/j.ahj.2010.01.015 20435182

[B44] Kagawa E Inoue I Kawagoe T Ishihara M Shimatani Y Kurisu S Nakama Y Dai K Takayuki O Ikenaga H Morimoto Y Ejiri K Oda N Assessment of outcomes and differences between in- and out-of-hospital cardiac arrest patients treated with cardiopulmonary resuscitation using extracorporeal life support Resuscitation 2010 81 968 973 10.1016/j.resuscitation.2010.03.037 20627526

[B45] Fox KA Dabbous OH Goldberg RJ Pieper KS Eagle KA Van de Werf F Avezum A Goodman SG Flather MD Anderson FA Jr Granger CB Prediction of risk of death and myocardial infarction in the six months after presentation with acute coronary syndrome: prospective multinational observational study (GRACE) BMJ 2006 333 1091 1096 10.1136/bmj.38985.646481.55 17032691 PMC1661748

[B46] Hamm CW Bassand JP Agewall S Bax J Boersma E Bueno H Caso P Dudek D Gielen S Huber K Ohman M Petrie MC Sonntag F Uva MS Storey RF Wijns W Zahger D Bax JJ Auricchio A Baumgartner H Ceconi C Dean V Deaton C Fagard R Funck-Brentano C Hasdai D Hoes A Knuuti J Kolh P ESC Committee for Practice Guidelines ESC Guidelines for the management of acute coronary syndromes in patients presenting without persistent ST-segment elevation: The Task Force for the management of acute coronary syndromes (ACS) in patients presenting without persistent ST-segment elevation of the European Society of Cardiology (ESC) Eur Heart J 2011 32 2999 3054 21873419 10.1093/eurheartj/ehr236

[B47] Belohlavek J Rohn V Tosovsky J Kunstyr J Semrad M Horak J Lips M Mlejnsky F Vykydal I Balík M Stritesky M Mrazek V Klein A Linhart A Lindner J A review of a newly established ECMO program in university affiliated cardiac center J Cardiovasc Surg 2011 52 445 451 21577197

[B48] Kunstyr J Lipy M Belohlavek J Prskavec T Mlejnsky F Koucky M Sebron V Balik M Spontaneous delivery during veno-venous extracorporeal membrane oxygenation in swine influenza-related acute respiratory failure Acta Anaesthesiol Scand 2010 54 1154 1155 10.1111/j.1399-6576.2010.02300.x 20887420

[B49] Belohlavek J Rohn V Jansa P Tosovsky J Kunstyr J Semrad M Horak J Lips M Mlejnsky F Balik M Klein A Linhart A Lindner J Veno-arterial ECMO in severe acute right ventricular failure with pulmonary obstructive hemodynamic pattern J Invasive Cardiol 2010 22 365 369 20679672

[B50] Rohn V Spacek M Belohlavek J Tosovsky J Cardiogenic Shock in Patient with Posterior Postinfarction Septal Rupture-Successful Treatment with Extracorporeal Membrane Oxygenation (ECMO) as a Ventricular Assist Device J Card Surg 2009 24 435 436 10.1111/j.1540-8191.2008.00710.x 18778295

[B51] Belohlavek J Mlcek M Huptych M Svoboda T Havranek S Ostadal P Boucek T Kovarnik T Mlejnsky F Mrazek V Belohlavek M Aschermann M Linhart A Kittnar O Coronary versus carotid blood flow and coronary perfusion pressure in a pig model of prolonged cardiac arrest treated by different modes of venoarterial ECMO and intraaortic balloon counterpulsation Crit Care 2012 16 R50 10.1186/cc11254 22424292 PMC3964801

[B52] Pokorna M Necas E Skripsky R Kratochvil J Andrlik M Franek O How accurately can the aetiology of cardiac arrest be established in an out-of-hospital setting? Analysis by “concordance in diagnosis crosscheck tables” Resuscitation 2011 82 391 397 10.1016/j.resuscitation.2010.11.026 21236546

